# Empathizing associates with mean diffusivity

**DOI:** 10.1038/s41598-019-45106-1

**Published:** 2019-06-20

**Authors:** Hikaru Takeuchi, Yasuyuki Taki, Rui Nouchi, Ryoichi Yokoyama, Yuka Kotozaki, Seishu Nakagawa, Atsushi Sekiguchi, Kunio Iizuka, Yuki Yamamoto, Sugiko Hanawa, Tsuyoshi Araki, Carlos Makoto Miyauchi, Kohei Sakaki, Yuko Sassa, Takayuki Nozawa, Shigeyuki Ikeda, Susumu Yokota, Magistro Daniele, Ryuta Kawashima

**Affiliations:** 1 Division of Developmental Cognitive Neuroscience, Institute of Development, Aging and Cancer, Tohoku University, Sendai, Japan; 2 Department of Behavioral Medicine, National Institute of Mental Health, National Center of Neurology and Psychiatry, Tokyo, Japan; 3 Department of Radiology and Nuclear Medicine, Institute of Development, Aging and Cancer, Tohoku University, Sendai, Japan; 4 Creative Interdisciplinary Research Division, Frontier Research Institute for Interdisciplinary Science, Tohoku University, Sendai, Japan; 5 Human and Social Response Research Division, International Research Institute of Disaster Science, Tohoku University, Sendai, Japan; 6 Advanced Brain Science, Institute of Development, Aging and Cancer, Tohoku University, Sendai, Japan; 7 School of Medicine, Kobe University, Kobe, Japan; 8 Division of Clinical research, Medical-Industry Translational Research Center, Fukushima Medical University School of Medicine, Fukushima, Japan; 9 Department of Functional Brain Imaging, Institute of Development, Aging and Cancer, Tohoku University, Sendai, Japan; 10 Department of Psychiatry, Tohoku Pharmaceutical University, Sendai, Japan; 11 Department of Psychiatry, Tohoku University Graduate School of Medicine, Sendai, Japan; 12 Department of Language Sciences, Graduate School of Humanities, Tokyo Metropolitan University, 192-0397 Tokyo, Japan; 13 Happiness Co-creation Society through “Ishin-Denshin” Intelligent Communications, Tokyo Institute of Technology, Tokyo, Japan; 14 Department of Ubiquitous Sensing, Institute of Development, Aging and Cancer, Tohoku University, Sendai, Japan; 15 School of Sport, Exercise, and Health Sciences, Loughborough University, Loughborough, England; 16 National Centre for Sport and Exercise Medicine (NCSEM), Loughborough University, Loughborough, England

**Keywords:** Attention, Personality

## Abstract

Empathizing is defined as “*the drive to identify another’s mental states and to respond to these with an appropriate emotion*” and systemizing is defined as “*the drive to the drive to analyze and construct rule-based systems*”. While mean diffusivity (MD) has been robustly associated with several cognitive traits and disorders related with empathizing and systemizing, its direct correlation with empathizing and systemizing remains to be investigated. We undertook voxel-by-voxel investigations of regional MD to discover microstructural correlates of empathizing, systemizing, and the discrepancy between them (D score: systemizing − empathizing). Whole-brain analyses of covariance revealed that across both sexes, empathizing was positively correlated with MD of (a) an anatomical cluster that primarily spreads in the areas in and adjacent to the left dorsolateral prefrontal cortex, left anterior to the middle cingulate cortex, and left insula and (b) an anatomical cluster of the left postcentral gyrus and left rolandic operculum. The former overlaps with positive MD correlates of cooperativeness. The D score and systemizing did not show significant correlations. In conclusion, while increased MD has generally been associated with reduced neural tissues and possibly area function, higher empathizing and cooperativeness were commonly reflected by greater MD values in areas (a) that mainly overlap with areas that play a key role in emotional salience and empathy. In addition, higher empathizing was correlated with greater MD values in areas (b) that play a key role in the mirror neuron system.

## Introduction

Empathizing and systemizing are important cognitive traits as stronger systemizing and weaker^1,2^ empathizing characterize thinking patterns of males and individuals with autism spectrum conditions (ASCs)^3,4^. Empathizing is “*the drive to identify another’s mental states and to respond to these with an appropriate emotion*”^1^. On the other hand, systemizing is “*the drive to the drive to analyze and construct rule-based systems*”^2^. The D score is the discrepancy between systemizing and empathizing (systemizing–empathizing) and characterizes thinking patterns of males and ASCs^5^. Among the cognitive characteristics of ASCs, social cognition deficits such as those related to the theory of mind are believed to be associated with a lower level of empathizing^6^, whereas higher competence in engineering, math, physics, and spatial cognition are believed be associated with higher systemizing^1,5^.

Previously, we investigated regional gray and white matter volume (rGMV and rWMV, respectively) and white matter structural connectivity (fractional anisotropy: FA) associated with empathizing, systemizing, and D score^7,8^. In these studies, we hypothesized that empathizing was associated with the default mode network (DMN) and systemizing was associated with the external attention system (EAS). EAS is the network that is active during the externally directed attention-demanding task and consists of inferior parietal lobes, the dorsal part of the anterior cingulate cortex (ACC) and lateral prefrontal cortices (LPFCs) and so on^9,10^. DMN is a network that is deactivated during these tasks and is recruited during socially-related cognition. This network includes, the superior temporal sulcus, some areas within the lateral temporal cortex, areas within the posterior cingulate cortices, precuneus, and medial prefrontal cortices (mPFCs)^9^. The results were more complicated, for example, in the analyses of rWMV, despite little correlation between empathizing and systemizing, positive rWMV correlates of empathizing and negative rWMV correlates of systemizing substantially overlapped and were located in the white matter, adjacent to the DMN and other networks, such as the ventral medial prefrontal cortex, the right inferior frontal gyrus, bilateral temporal lobe, and the posterior cingulate cortex^7^. Furthermore, negative rGMV correlates of empathy were located not only in DMN areas such as the mPFC, precuneus, middle cingulate gyrus, and temporal pole but also in EAS areas such as the LPFC, superior parietal lobule, and ACC and subcortical areas such as the thalamus and the caudate. A significant positive correlation was found between systemizing and rGMV in the LPFC—the key node of EAS—as well as a negative correlation with the putamen and caudate^8^.

On the other hand, mean diffusivity (MD) of diffusion tensor imaging (DTI)^11^ measures microstructural brain properties. As we summarized previously^12^, lower MD is caused by “*a greater density of cellular structures in tissues, such as capillaries, synapses, and macromolecular proteins,”* as well as *“changes in the shapes of neurons or glia and the directionality of tissue organization (e.g., by strengthening of the axonal or dendritic backbones and the surrounding tissues)*”^11–13^. Therefore, an MD decrease is generally considered to reflect local functional augmentation, and higher individual cognitive competence is usually associated with a lower MD of the relevant areas^14^. Several studies have demonstrated a robust and characteristic correlation between the MD measurements of gray and white matter and individual cognitive differences, compared with volume and fractional anisotropy measures of DTI (fractional anisotropy reflects the myelination of white matter, the properties of the axon, the direction of tracts, etc)^12,15,16^. In addition, autistic subjects have been shown to exhibit a robustly elevated MD in extensive regions in the brain, which is suggestive of this measure’s relevance in autism^17^. Reduction, however, of certain tissue components such as synapses is associated with functional refining, whereas both greater and lesser cortical thickness and rGMV are associated with greater cognitive competence, depending on the conditions^8,14^. Similarly, an MD increase may be associated with a greater cognitive competence. Consistently, our previous study showed that the personality trait of cooperativeness, which is associated with social competence, was positively correlated with MD in areas close to the ACC, insula, and LPFC^16^.

Despite these data, MD correlates (including those of gray and white matter) of empathizing, systemizing, and D score have never been investigated. The purpose of this study was to investigate these issues. Given the importance of these psychological measures in ASDs and the unique ability of MD to reveal the neural bases of individual cognitive differences, it is vital to understand the association between MD and empathizing/systemizing/D score.

Based on the abovementioned background we set two hypotheses. One was that MD of DMN would be associated with empathizing and MD of EAS would be associated with systemizing. The other was that empathizing would positively correlate with MD in the areas between ACC and LPFC (the key node of EAS), similar to cooperativeness, which shares prosocial components with empathizing.

## Methods

### Subjects

The present study is a part of an ongoing project aiming to investigate the association between brain imaging, cognitive function, and aging. The descriptions in this subsection have been reproduced from our previous study, in which the exact same methods were used^7,16,18–20^. It included EQ, SQ measures and imaging data from 1332 healthy, right-handed individuals (774 men and 558 women). The mean age of the subjects was 20.8 years [standard deviation (SD), 1.8; age range: 18–27 years old]. The following descriptions were mostly reproduced from another study of ours from the same project using the exactly same methods regarding these issues^21^. All subjects were university students, postgraduates, or university graduates of less than one year’s standing. All subjects had normal vision and none had a history of neurological or psychiatric illness. Handedness was evaluated using the Edinburgh Handedness Inventory^22^.

Among the subjects of this study, data from 567 subjects were used in our previous study investigating the associations between empathizing/systemizing and rGMV^8^ and between FA and rWMV^7^, and data from 248 subjects were previously used to investigate the association between empathizing/systemizing and resting state functional connectivity^23^. Among the subjects of the present study, several participated also in our intervention studies (psychological data and imaging data recorded before the intervention were used in this study)^24^. Psychological tests and MRI scans not described in this study were performed together with those described in this study. The subjects were recruited by advertising the study on the bulletin boards of the Tohoku University or by emailing the information to potential subjects. Written informed consent was obtained from each subject. For nonadult subjects, written informed consent was obtained from their parents (guardians). This study was approved by the Ethics Committee of Tohoku University.

For the day of the cognitive tests and MRI scans, the subjects were instructed to get sufficient sleep, maintain their normal conditions, eat sufficient breakfast, and consume their usual amount of caffeinated foods and drinks. In addition, they were instructed to avoid alcohol on the night before the assessment.

### Systemizing quotient (SQ) and empathy quotient (EQ) questionnaires

Japanese versions^25^ of the SQ and EQ questionnaires^3,4^ were administered. The following methods were reproduced from our previous study using the exact same method^7,8,23,26^. The EQ score was used as an index of empathizing, and the SQ score was used as an index of systemizing. These tests consist of 40 items for each quotient and 20 filler items that are not scored. The scales consist of self-descriptive statements scored on a four-point scale ranging from Strongly Disagree to Strongly Agree. Half the items are worded to produce an “agree” response and rest to produce a “disagree” response. Items are randomized to avoid a response bias. Each strong systemizing/empathizing response is awarded 2 points, and each slightly systemizing/empathizing response is awarded 1 point (i.e., each item is scored as 2, 1, or 0), resulting in a range of total scores from 0–80 for each quotient.

The D score was calculated according to a previous study^27^. The raw SQ and EQ scores were standardized by subtracting the population mean from the score then dividing it by the maximum possible score: S = (raw SQ score − population mean of the raw SQ score)/80 and E = (raw EQ score − population mean of the raw EQ score)/80. For this computation, we used the estimated population means (EQ: mean = 33.4, SQ: mean = 22.7 within the whole sample) derived from a previous study’s large sample (N = 1250) of Japanese university students with an almost equal number of men and women^25^. This procedure was performed according to our previous studies^8^. The discrepancy between systemizing and empathizing was then quantified as D = (S − E)/2. The greater the D score in a positive direction, the stronger one’s systemizing relative to one’s empathizing. D scores close to zero represent an equal drive to systemize and empathize.

The questionnaire comprised the psychometric properties described below. Some studies have reported empathizing and systemizing as largely independent. However, a weak negative correlation between them has been reported by several studiese.g.^28^, whereas others failed to find such correlatione.g.^25^. Individuals with autism spectrum conditions have been found to exhibit higher SQ scores and lower EQ scores than controls^25^. Similarly, male individuals exhibit higher SQ scores than female individuals who, in turn, present higher EQ scores than males^29^. Students of humanities also show higher EQ scores than students of science who, in turn, present higher SQ scores than those studying humanities^29^. Additionally, actors were found to have higher EQ scores^30^. EQ is positively correlated with both the size of an individual’s social network^31^ and their performance on a face perception task^32^. The Autism Spectrum Quotient (AQ) is a measure of autistic traits. Although that measure was not collected in this project, it is well explained by the model including both EQ and SQ (more than 75% of the variance)^28^, whereas the AQ score is strongly and significantly correlated with the D score (r = 0.69)^33^. These findings have demonstrated the criterion-related validity of the present questionnaire. The internal consistencies of both EQ and SQ, calculated in a previous, large sample study, were 0.86 and 0.88, respectively, demonstrating the reliability of this questionnaire.

The Japanese version of the questionnaires was validated by Prof. Akio Wakabayashi, Prof. Baron-Cohen, and others^25,34^. In the Japanese version, the patterns of male EQ and SQ scores (vs. female EQ and SQ scores), ASC group (vs. controls), and science majors (vs. humanities majors) were similar to those of the original version^25,34^. The present study’s participants’ EQ score was lower than that of the previous study’s control sample^3,4^, but similar to the EQ scores shown by the previous study’s Japanese university students^25,34^.

The following are examples of items found on the SQ–EQ questionnaires:

“I can tune into how someone else feels rapidly and intuitively” (EQ)

“I am good at predicting how someone will feel” (EQ)

“I am fascinated by how machines work” (SQ)

“If I were buying a stereo, I would want to know about its precise technical features” (SQ)

The timing for the subjects to answer the questionnaires was not fixed along the project but was set within two months before or after MRI scans, except in rare cases, such as that of subjects having to postpone the experiments for a while due to illness. Since empathizing and systemizing are individual traits, they are not supposed to be influenced by the timing.

### Assessment of psychometric measures of general intelligence

Raven’s Advanced Progressive Matrix^35^, which is often shown to be the measure most correlated with general intelligence and thus the best measure of general intelligence^35^, was used to assess intelligence and adjust for the effect of general intelligence on MD. For additional details on administration of Raven’s Advanced Progressive Matrix, refer to our previous studies^36,37^. The descriptions in this subsection were mostly reproduced from our previous study using the exact same method^7^.

### Assessment of cooperativeness

To measure cooperativeness, we used a Japanese version^38^ of the Temperament Character Inventory^39^.

### Image acquisition

MRI data acquisition was performed using a 3T Philips Achieva scanner. The descriptions in this subsection have been mostly reproduced from our previous study that used the exact same methods^40^. All data was obtained in our facility, using a single scanner (Institute of Development, Aging and Cancer, Tohoku University). Diffusion-weighted data were acquired using a spin-echo EPI sequence (TR = 10293 ms, TE = 55 ms, big delta (Δ) = 26.3 ms, little delta (δ) = 12.2 ms, FOV = 22.4 cm, 2 × 2 × 2 mm^3^ voxels, 60 slices, SENSE reduction factor = 2, number of acquisitions = 1). The diffusion weighting was isotropically distributed along 32 directions (*b* value = 1,000 s/mm^2^). Additionally, three images with no diffusion weighting (*b* value = 0 s/mm^2^) (b = 0 images) and one b = 0 image were acquired from 1207 and 125 subjects, respectively, using a spin-echo EPI sequence (TR = 10293 ms, TE = 55 ms, FOV = 22.4 cm, 2 × 2 × 2 mm^3^ voxels, 60 slices). When three b = 0 images were obtained, the average of the three images was generated in the console and used for the following preprocessing procedure. From the collected images, FA maps and MD maps were calculated using the commercially available diffusion tensor analysis package on the MR consol. For more details, see Supplemental Methods.

### Preprocessing of imaging data

Preprocessing and analysis of imaging data were performed using SPM8 implemented in Matlab. The descriptions in this subsection have been mostly reproduced from our previous study that used the exact same methods^41^. Basically, we normalized MD images of subjects with previously validated^7^ diffeomorphic anatomical registration through exponentiated lie algebra (DARTEL)-based registration process method to give images with 1.5 × 1.5 × 1.5 mm^3^ voxels, then tissues that are not likely to be gray or white matter were carefully removed and smoothed by convolving them with an isotropic Gaussian kernel of 8-mm full width at half maximum. For details, see Supplemental Methods.

### Statistical analysis of MD

In the whole brain analyses, we used voxel-wise analysis of covariance (ANCOVA), with sex difference as a grouping factor (using the full factorial option of SPM). The descriptions in this subsection were mostly reproduced from our previous study using the same method^7,8,23,41^.

In D score analyses, age, RAPM score, the number of b = 0 images, and D score were covariates. In the analyses of the EQ and SQ scores, age, RAPM score, the number of b = 0 images, EQ score, and SQ score were covariates. In analyses of cooperativeness, age, RAPM score, and cooperativeness were covariates (in the sample from which the cooperativeness score was gathered, single b = 0 images were obtained from few subjects and these subjects were excluded in the analyses of the cooperativeness score). We performed three different whole-brain ANCOVAs.

In these analyses, age, RAPM score, and target variables (D/EQ/SQ/cooperativeness scores) were modeled so that each covariate had a unique relationship with MD for each sex (using the interactions option in SPM), which enabled investigation of the effects of interactions between sex and each covariate. On the other hand, the number of b = 0 images was not modeled in this manner, and a common effect of the number of b = 0 images on MD was assumed for both sexes (in analyses of cooperativeness, this covariate did not exist). In these analyses, the centering option was used for centering the all covariates. The main effects of the target variables (D/EQ/SQ/cooperativeness scores) (contrasts of [the effects of the target variables (D/EQ/SQ/cooperativeness scores) for males and females] were [1 1] or [−1 −1]) and the interaction between sex and the target variables (D/EQ/SQ/cooperativeness scores) (contrasts of [the effect of the target variables (D/EQ/SQ/cooperativeness scores) for males, the effect of the target variables (D/EQ/SQ/cooperativeness scores) for females] were [−1 1] or [1 −1]) were assessed using t-contrasts. Analysis was limited to the gray and white matter masks, which comprise areas highly likely to be gray or white matter (as described in Supplemental Methods**)**.

Sex differences in the MD correlates of empathizing and systemizing were investigated, as a previous study showed both commonalities and differences in the brain structural and functional connectivity correlates of empathizing^7,8,23^. General intelligence control is a standard procedure and general intelligence is included as a covariate to exclude the possibility of associations between MD and empathizing or systemizing, explained in terms of the associations between general intelligence and MD (combined with those between general intelligence and empathizing or systemizing).

The anatomical labels of significant clusters of major white matter fibers presented in the Results section were determined using the ICBM DTI-81 Atlas (http://www.loni.ucla.edu/).

A multiple comparison correction of the cross-sectional analyses was performed using threshold-free cluster enhancement (TFCE)^42^, with randomized (5,000 permutations) nonparametric permutation testing via the TFCE toolbox (http://dbm.neuro.uni-jena.de/tfce/). We applied the threshold of an FWE corrected *P* < 0.05.

### Ethical approval

This study was approved by the Ethics Committee of Tohoku University. All experiments were performed in accordance with declaration of Helsinki.

## Results

### Behavioral data

The mean EQ scores of males and females were 30.24 (SD, 9.64; range 7–66) and 34.68 (SD, 9.86; range 12–67), respectively. The mean SQ scores of males and females were 28.44 (SD, 8.59; range 6–57) and 21.60 (SD, 7.36; range 8–54), respectively. The mean D scores of males and females were 0.0556 (SD, 0.0698; range −0.1644 to 0.2981) and −0.0149 (SD, 0.0700; range −0.2206 to 0.1919), respectively. The mean, SD, and range of all psychological variables is presented in Table 1.

**Table 1:** Demographic variables of males and females included in our study.

Measure	Males			Females		
	Mean	SD	Range	Mean	SD	Range
Age	20.86	1.87	18–27	20.70	1.62	18–27
RAPM^a^	28.70	3.83	13–36	28.04	3.83	15–36
EQ	30.24	9.64	7–66	34.68	9.86	12–67
SQ	28.44	8.59	6–57	21.60	7.36	8–54
D score	0.0556	0.0698	−0.1644–0.2981	−0.0149	0.07	−0.2206–0.1919
Cooperativeness	26.35	5.88	8–41	28.21	6.1	5–40

^a^Raven’s Advanced Progressive Matrix.

For all subjects, simple regression analyses showed significant (a) positive correlation between EQ and SQ scores, (b) negative correlation between EQ and D scores, (c) negative correlation between EQ and RAPM scores, (d) positive correlation between EQ score and cooperativeness, (d) positive correlation between SQ and D scores, (e) positive correlation between SQ and RAPM scores, (f) positive correlation between D and RAPM scores, and (g) negative correlation between D score and cooperativeness.

For male subjects, simple regression analyses showed significant (a) positive correlation between EQ and SQ scores, (b) negative correlation between EQ and D scores, (c) positive correlation between SQ and D scores, (d) positive correlation between SQ and RAPM scores, (e) positive correlation between SQ score and cooperativeness, (f) positive correlation between D and RAPM scores, and (g) negative correlation between D score and cooperativeness.

For female subjects, simple regression analyses showed (a) positive correlation between EQ and SQ scores, (b) negative correlation between EQ and D scores, (c) negative correlation between EQ and RAPM scores, (d) positive correlation between EQ score and cooperativeness, (d) positive correlation between SQ and D scores, (e) positive correlation between SQ and RAPM scores, (f) positive correlation between D and RAPM scores, (g) negative correlation between D score and cooperativeness, and (h) negative correlation between RAPM and cooperativeness scores. For statistical values, see Table 2.

**Table 2:** Statistical results (standard beta coefficient, *t*-value, uncorrected *p*-values, and degree of freedom) of the simple correlation analyses performed on psychological variables.

	EQ score	SQ score	D score	RAPM score^a^	Cooperativeness
EQ score	—	—		—	
SQ score	MF: 0.117, 4.286, 1.90*10^−5^, 1331* M: 0.254, 7.294, 7.45*10^−13^, 773* F: 0.178, 4.263, 2.40*10^−5^, 557*	—			
D score	MF: −0.717, −37.470, 2.36*10^−210^, 1331* M: −0.668, −24.944, 3.48*10^−101^, 773* F: −0.763, −27.823, 2.16*10^−107^, 557*	MF: 0.609, 28.005, 4.80*10^−136^, 1331* M: 0.550, 18.302, 1.99*10^−62^, 773* F: 0.501, 13.632, 1.03*10^−36^, 557*	—		
RAPM score	MF: −0.091, −3.332, 8.85*10^−4^, 1331* M: −0.004, −0.111, 0.912, 773 F: −0.170, −4.063, 5.50*10^−5^, 557*	MF: 0.163, 6.030, 2.12*10^−9^, 1331* M: 0.168, 4.737, 3.00*10^−6^, 773* F: 0.100, 2.367, 0.0183, 557*	MF: 0.187, 6.951, 5.67*10^−12^, 1331* M: 0.133, 3.721, 2.13*10^−4^, 773* F: 0.215, 5.191, 2.94*10^−7^, 557*	—	
Cooperativeness	MF: 0.435, 16.840, 2.44*10^−57^, 1213* M: 0.428, 12.530, 1.22*10^−32^, 701* F: 0.404, 9.982, 1.49*10^−21^, 511*	MF: 0.019, 0.676, 0.499, 1213 M: 0.093, 2.477, 0.0135, 701* F: 0.074, 1.680, 0.0936, 511	MF: −0.338, −12.501, 8.15*10^−34^, 1213* M: −0.303, −8.409, 2.31*10^−16^, 701* F: −0.309, −7.325, 9.38*10^−13^, 511*	MF: −0.047, −1.635, 0.102, 1213 M: 0.007, 0.184, 0.854, 701 F:−0.089, −2.027, 0.0431, 511*	—

M: male; F: female; MF: all participants.

^a^Raven’s advanced progressive matrices (i.e., a general intelligence task).

*P < 0.05.

**Figure 1:**
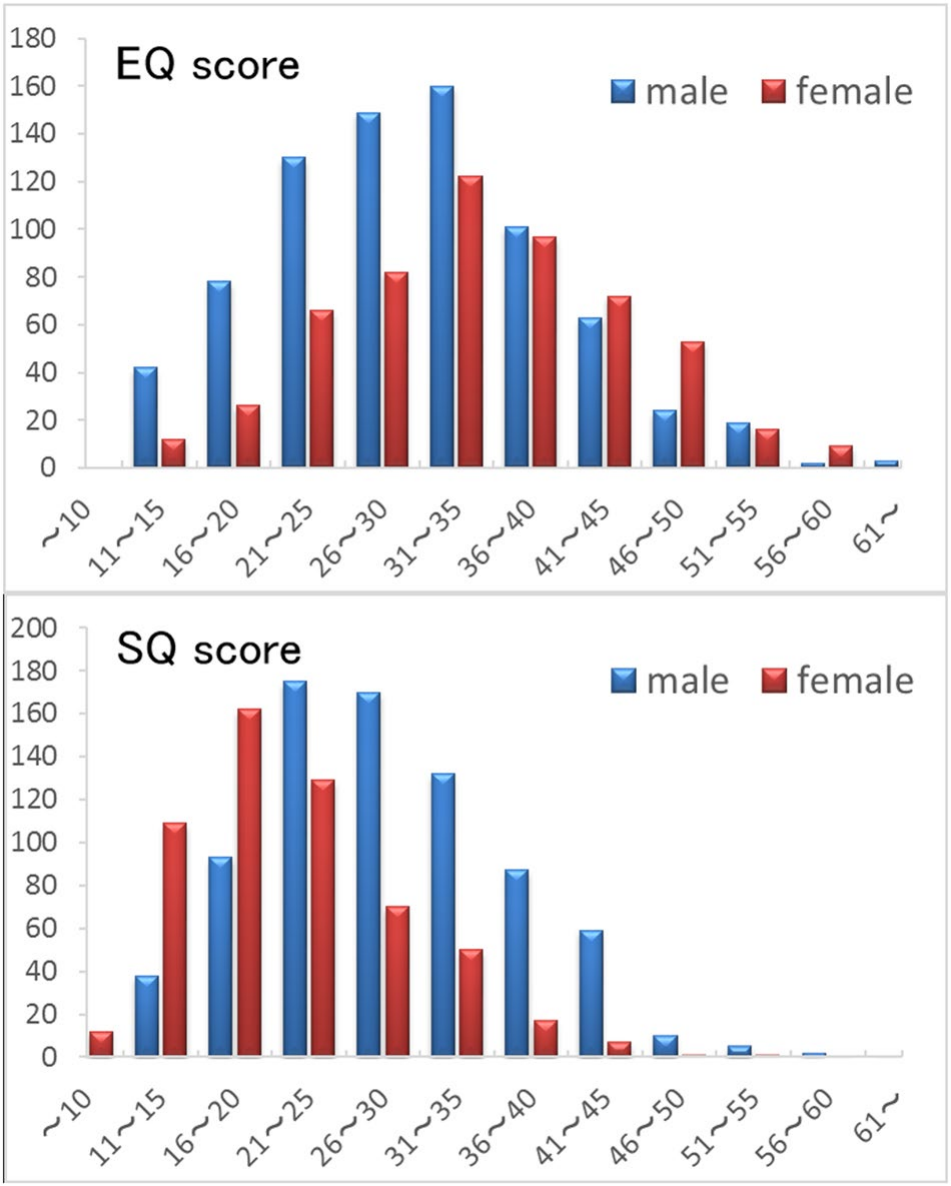
Histograms showing the EQ scores (**a**) and SQ scores (**b**) for all subjects.

**Figure 2:**
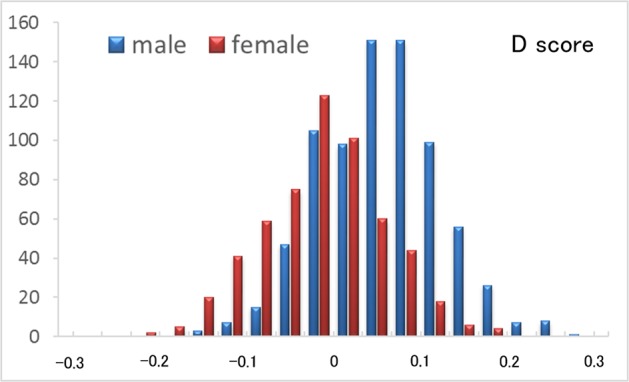
Histograms showing the D scores for all subjects.

Figure 1 presents the data of distributions of EQ and SQ scores. Figure 2 presents the data of distributions of D scores.

These results can be summarized as follows:

- In both sexes, D scores (discrepancy between systemizing and empathizing [systemizing − empathizing]) was positively correlated with SQ score and negatively correlated with EQ score, as expected from the definition of D score.
- In both sexes, cooperativeness was positively correlated with EQ score and negatively correlated with D score, which is consistent with the pro-social characteristics of cooperativeness as described in the Introduction.
- In both sexes, RAPM score and SQ score were positively correlated, consistent with the nature of systemizing (the drive to analyze the rules that govern a system)^1^.
- In both sexes, EQ score and SQ score were positively correlated, although only weakly so.
- In females, EQ score and cooperativeness were negatively correlated with RAPM score, suggesting sex-specific characteristics of empathizing and cooperativeness.

### Effects of EQ, SQ, and D scores on MD

**Figure 3:**
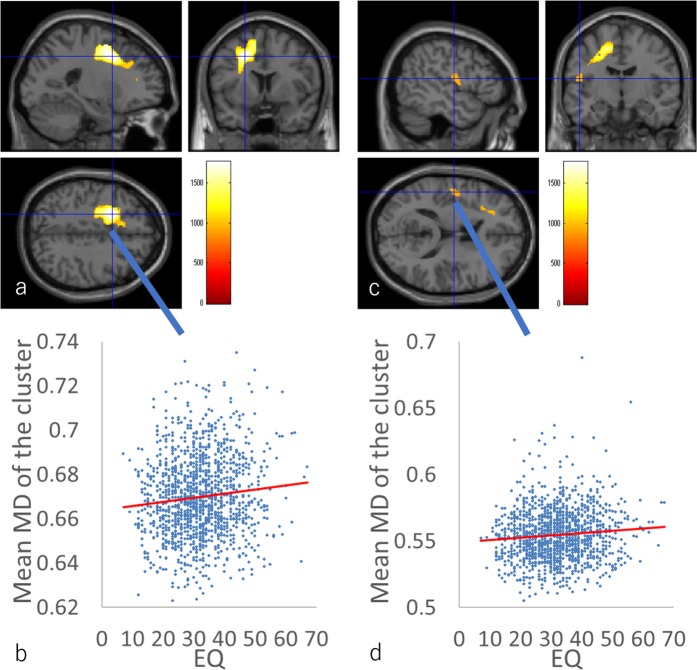
The main positive effects (regardless of sex) of the EQ score across both sexes. (**a**,**c**) The results shown were obtained using a threshold of threshold-free cluster enhancement (TFCE) of *P* < 0.05, based on 5000 permutations. The results were corrected at the whole-brain level. Regions with significant correlations are overlaid on a “single subject” T1 image of SPM8. The color represents the strength of the TFCE value. (**a**) Regions with negative main positive effects of EQ scores on MD across both sexes were observed in the areas in and adjacent to the left dorsolateral prefrontal cortex, left ACC, and left insula. (**b**) Scatter plot of the associations between EQ scores and mean MD values for this cluster of (**a**). (**c**) Regions with positive main positive effects of EQ scores on MD across both sexes were observed in the areas of the left postcentral gyrus and left rolandic operculum. (**d**) Scatter plot of the associations between EQ scores and mean MD values for this cluster of (**b**).

ANCOVA involving both EQ and SQ scores revealed a significant overall positive effect (regardless of sex) of the EQ score on MD in the anatomical cluster that spreads in the areas in and adjacent to the left dorsolateral prefrontal cortex, left ACC, and the left insula; in the anatomical cluster of the left postcentral gyrus and left rolandic operculum; and in the anatomical cluster of the left middle cingulate gyrus (Fig. 3, Table 3). There were no other significant effects of EQ and SQ scores, interaction between sex and the EQ score, and interaction between sex and the SQ score on MD. ANCOVA involving the D score revealed no significant effects of the D score or of the interaction between D score and sex on MD.

**Table 3:** Brain regions that exhibited significant positive correlations between empathizing and MD.

Included gray matter areas* (number of significant voxels in the left and right side of each anatomical area)	Included large bundles** (number of significant voxels in the left and right side of each anatomical area)	x	y	z	TFCE value	Corrected *p* value (FWE)	Cluster size (voxel)	r***
Anterior cingulum (L:1)/Middle cingulum (L:185)/Inferior frontal operculum (L:10)/Inferior frontal triangular (L:64)/Middle frontal other areas (L:848)/Superior frontal medial area (L:76)/Superior frontal other areas (L:647)/Insula (L:6)/Precentral gyrus (L:137)/Supplemental motor area (L:763)/	Body of corpus callosum (1)/Anterior corona radiata (L:169)/Superior corona radiata (L:534)/Superior longitudinal fasciculus (L:79)/	−22.5	7.5	42	1764	0.004	6091	0.102
Postcentral gyrus (L:56)/Rolandic operculum (L:123)/	Superior longitudinal fasciculus (L:3)/	−49.5	−10.5	15	944	0.0442	172	0.093
Middle cingulum (L:1)/	None	−4.5	22.5	37.5	917	0.0486	1	0.055

^*^Labelings of the anatomical regions of gray matter were based on the WFU PickAtlas Tool (http://www.fmri.wfubmc.edu/cms/software#PickAtlas/)^70,71^ and on the PickAtlas automated anatomical labeling atlas option^72^. Temporal pole areas included all subregions in the areas of this atlas.

**The anatomical labels and significant clusters of major white matter fibers were determined using the ICBM DTI-81 Atlas (http://www.loni.ucla.edu/).

***Simple correlation coefficients between the mean values of the significant clusters and those of the empathizing score. Note that due to whole-brain analyzes overfitting^69^, the correlation coefficients of significant areas are overestimated to a degree dependent on the sample size and number of comparisons.

### Effects of cooperativeness on MD

**Figure 4:**
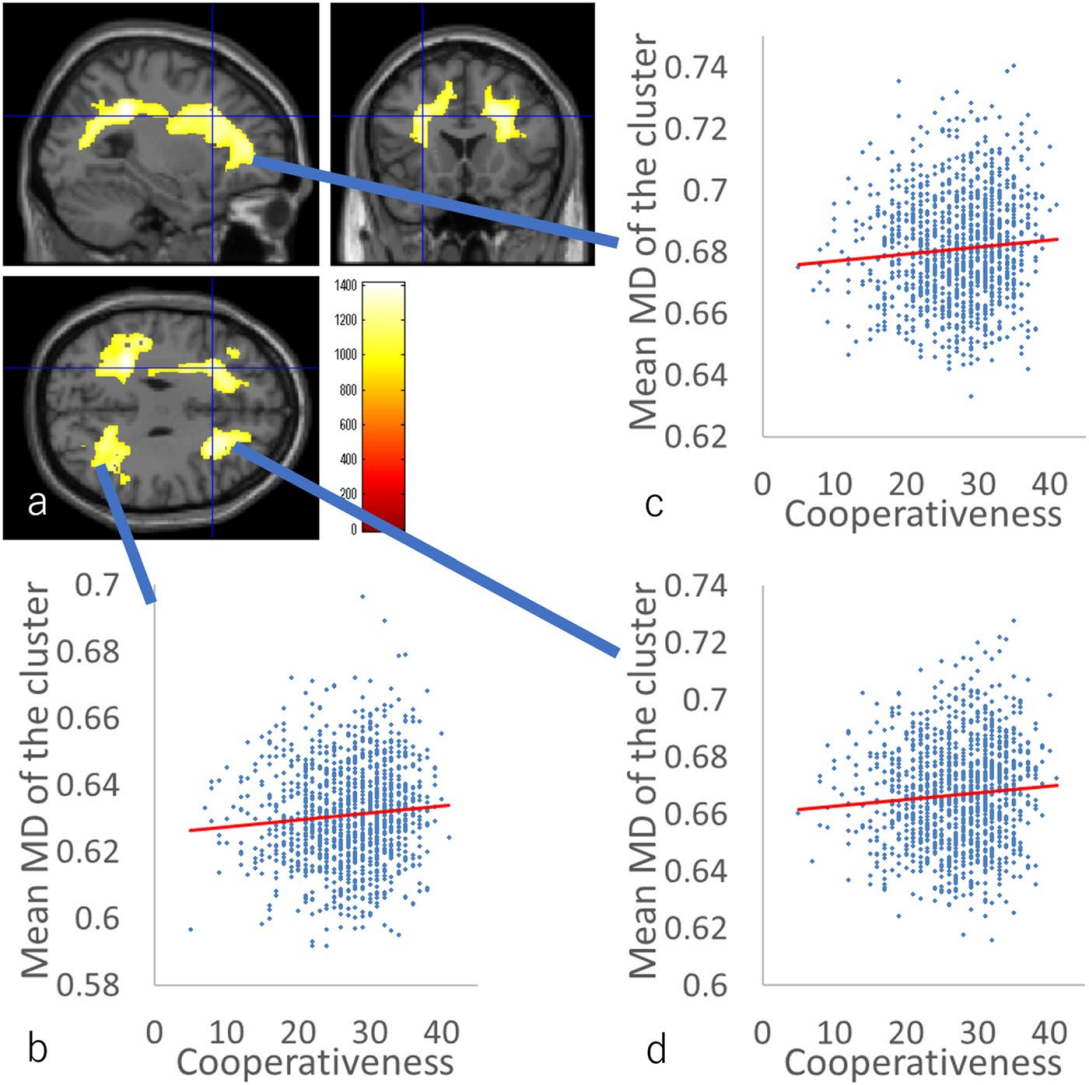
The main positive effects (regardless of sex) of cooperativeness score across both sexes. (**a**) The results shown were obtained using a threshold of threshold-free cluster enhancement (TFCE) of *P* < 0.05, based on 5000 permutations. The results were corrected at the whole brain level. Regions with significant correlations are overlaid on a “single subject” T1 image of SPM8. The color represents the strength of the TFCE value. Regions with negative main positive effects of cooperativeness scores on MD across both sexes were observed in the areas in and adjacent to the gray and white matter areas of bilateral frontal and parietal areas. (**b**–**d**) Scatter plot of the associations between EQ scores and mean MD values for the cluster spreading mainly across the posterior areas of the right hemisphere (**b**), mean MD values for the cluster spreading mainly across the left hemisphere (**c**), mean MD values for the cluster spreading mainly across the anterior areas of the right hemisphere (**d**).

ANCOVA involving cooperativeness revealed a significant overall positive effect (regardless of sex) of the cooperativeness on MD in the anatomical cluster that spreads in the areas in and adjacent to the left dorsolateral prefrontal cortex, left ACC, and the left insula; in the anatomical cluster that spreads in the areas in and adjacent to the right dorsolateral prefrontal cortex, the right ACC, and right insula; in the anatomical cluster that spreads in and adjacent to the left lateral and medial parietal lobes as well as in the anatomical cluster that spreads in and adjacent to the right lateral and medial parietal lobes (Fig. 4, Table 4).

**Table 4:** Brain regions that exhibited significant positive correlations between cooperativeness and MD.

Included gray matter areas* (number of significant voxels in the left and right side of each anatomical area)	Included large bundles** (number of significant voxels in the left and right side of each anatomical area)	x	y	z	TFCE value	Corrected *p* value (FWE)	Cluster size (voxel)	r***
Angular gyrus (L:237)/Calcarine Cortex (L:40)/Anterior cingulum (L:24)/Middle cingulum (L:74)/Posterior cingulum (L:12)/Inferior frontal operculum (L:8)/Inferior frontal orbital area (L:135)/Inferior frontal triangular (L:492)/Middle frontal orbital area (L:2)/Middle frontal other areas (L:444)/Superior frontal medial area (L:100)/Superior frontal other areas (L:462)/Insula (L:191)/Middle occipital lobe (L:168)/Superior occipital lobe (L:26)/Inferior parietal lobule (L:204)/Superior parietal lobule (L:136)/Postcentral gyrus (L:57)/Precentral gyrus (L:97)/Precuneus (L:215)/Rolandic operculum (L:165)/Supplemental motor area (L:146)/Supramarginal gyrus (L:457)/Middle temporal gyrus (L:41)/Superior temporal gyrus (L:249)/	Genu of corpus callosum (25)/Body of corpus callosum (27)/Splenium of corpus callosum (8)/Anterior limb of internal capsule (L:3)/Posterior limb of internal capsule (L:6)/Retrolenticular part of internal capsule (L:1)/Anterior corona radiata (L:1247)/Superior corona radiata (L:1110)/Posterior corona radiata (L:682)/Posterior thalamic radiation (L:157)/External capsule (L:29)/Superior longitudinal fasciculus (L:597)/Superior fronto-occipital fasciculus (L:14)/Tapatum (L:9)/	−31.5	−42	34.5	1410	0.0148	12276	0.082
Caudate (R:14)/Anterior cingulum (R:145)/Middle cingulum (R:34)/Inferior frontal operculum (R:99)/Inferior frontal orbital area (R:92)/Inferior frontal triangular (R:133)/Middle frontal medial area (R:40)/Middle frontal orbital area (R:11)/Middle frontal other areas (R:627)/Superior frontal medial area (R:125)/Superior frontal orbital area (R:46)/Superior frontal other areas (R:855)/Insula (R:4)/Supplemental motor area (R:50)/	Anterior corona radiata (R:1062)/Superior corona radiata (R:57)/Superior longitudinal fasciculus (R:2)/	24	21	33	1312	0.0182	6033	0.078
Angular gyrus (R:458)/Calcarine Cortex (R:347)/Middle cingulum (R:40)/Cuneus (R:90)/Middle occipital lobe (R:465)/Superior occipital lobe (R:116)/Inferior parietal lobule (R:33)/Superior parietal lobule (R:59)/Precuneus (R:493)/Supramarginal gyrus (R:85)/Middle temporal gyrus (R:275)/Superior temporal gyrus (R:13)/	Splenium of corpus callosum (35)/Posterior corona radiata (R:205)/Posterior thalamic radiation (R:243)/Superior longitudinal fasciculus (R:138)/	18	−54	36	1230	0.0232	5056	0.088
Lingual gyrus (R:38)/	None	16.5	−85.5	−7.5	938	0.0494	38	0.052

^*^Labeling of the anatomical regions of gray matter were based on the WFU PickAtlas Tool (http://www.fmri.wfubmc.edu/cms/software#PickAtlas/)^70,71^ and the PickAtlas automated anatomical labeling atlas option^72^. Temporal pole areas included all sub-regions in the areas of this atlas.

**The anatomical labels and significant clusters of major white matter fibers were determined using the ICBM DTI-81 Atlas (http://www.loni.ucla.edu/).

***Simple correlation coefficients between the mean values of the significant clusters and the cooperativeness score. Note that due to whole-brain analyzes overfitting^69^, the correlation coefficients of significant areas are overestimated to a degree dependent on the sample size and number of comparisons.

There was a substantial overlap between the cluster of overall positive effects of the EQ score and the cluster of overall positive effects of cooperativeness in the areas in and adjacent to the gray and white matter areas of the left dorsolateral prefrontal cortex, left ACC, and left insula.

## Discussion

To the best of our knowledge, this is the first study to successfully reveal MD correlates of empathizing. Our results showed that both sexes present a positive correlation between empathizing and the MD of an anatomical cluster primarily adjacent to the left dorsolateral prefrontal cortex, left anterior and middle cingulate cortex, and left insula; the anatomical cluster of the left postcentral gyrus and left rolandic operculum; and the anatomical cluster of the left middle cingulate gyrus. Further, cooperativeness is positively correlated with MD of the bilateral anatomical clusters that spread between the anterior and middle cingulate cortex, medial prefrontal cortex, insula lateral prefrontal cortex as well as the bilateral anatomical clusters that spread between the medial parietal cortex and lateral parietal cortex. These data support our second hypothesis that predicted an overlap between MD correlates of empathizing and those of cooperativeness in the area between the dorsal part of the ACC, the insula, and the lateral prefrontal cortex. However, the hypothesized involvement of DMN areas in empathizing and the involvement of EAS areas in systemizing were not supported, suggesting that MD correlates have unique characteristics. The results suggest that empathizing is reflected in microstructural properties in areas that overlap with areas that play key roles in emotional salience and empathy as well as with areas that play key roles in the mirror neuron system, as discussed below.

Overall, previous studies have generally suggested that increased MD is associated with reduced neural tissues and possibly function. As described similarly in our previous study^15^, decreased MD has been suggested to reflect various cellular and cytoarchitectonic changes resulting in higher tissue density in various tissue components, such as synapses, macromolecular proteins, capillaries, and spines; changes in the properties of myelin, axon and membrane; shape alterations of glia or neurons; or enhanced tissue organization^11,13^. However, MD is not specifically sensitive to any one of them^11,13^. Therefore, MD decrease is thought to reflect tissue and functional adaptation increase^14^. Consistently, a greater motivational state is associated with lower MD of the subcortical areas involved in motivation, such as the putamen and pallidum^15^, and greater performance IQ is associated with lower MD of the extensive areas across the whole brain areas^14^. However, lower MD can indicate blood flow decreases and in certain cases, functional adaptation is seemingly reflected in an increase in MD^43^. Therefore, whether lower MD signifies an adaptive condition cannot be definitely concluded. In addition, while in autistic subjects, MD is robustly and extensively elevated and ASCs is characterized with lower empathizing^17^, in this study, lower empathizing is associated with lower MD. Therefore, the neural mechanisms behind the variations in tendencies of ASCs in the normal sample and the neural mechanisms behind autism may be different.

There was an overlap of positive MD correlates of empathizing and cooperativeness in the anatomical cluster between the dorsal part of ACC, the lateral prefrontal cortex, and the insula. The anterior insula and the dorsal-anterior/anterior-midcingulate cortex play central roles in responses in the domain of various pleasant and disgusting feelings. Moreover, those regions play a central role in the subjective experiences and adaptive responses toward the predicted and actual states, both those within oneself and in others. Empathy constitutes a special case among these general cognitive processes^44,45^. Therefore, augmentation of these regional functions is possibly reflected in greater MD due to increased default cerebral blood flow. Other mechanisms may contribute to empathizing and they form the observed correlations herein. Alternatively, the anterior cingulate and anterior insula form the salience network^46^, which is thought to integrate interoceptive information with emotional salience^46,47^. Greater functioning of these areas is thought to be associated with generalized anxiety^46^ and social anxiety^48^. Thus, as we noted previously^16^, we speculate that a greater amount of tissues in this pathway may be associated with social anxiety, which in turn may prohibit prosociality that likely plays a key role in empathizing and cooperativeness. However, because this overlap of MD correlates was extensive, and this area is adjacent to other networks, such as EAS, positive MD correlates in this area may reflect other cognitive factors, such as relatively weakened function of EAS, or increased cerebral blood flow to these areas. Therefore, future studies need to elucidate the nature of increased MD in this area. Furthermore, in relation to the observed findings in the insula, a previous study has shown that a greater score in a measure of affective empathy (personal distress, defined as a focus on having aversive emotional feelings when witnessing another’s pain or anguish) was associated with a greater magnetization transfer measure^49^. This measure captures not only the effects of macromolecules, predominantly myelin content, but also the effects of other cell components that facilitate myelination and overall myeloarchitectural integrity^49^. Since a lower measure of magnetization transfer and a greater MD are associated with advanced aging in similar areas^50^, the present findings regarding a positive correlation between empathizing and MD in the insular and contingent areas may be parallel to the previous findings regarding a negative correlation between a measure of affective empathy and the magnetization transfer measure.

There was no significant correlation between MD and systemizing. Our previous study showed that among seven major personalities of temperament and character inventory, cooperativeness showed positive associations with MD in bilateral areas between ACC, lateral prefrontal cortex, and insula^16^. However, other six personalities that showed substantial correlation with cognitive components of motivation were correlated with MD measurements in limited areas related to the dopaminergic system, including the putamen, pallidum, caudate and as well as contingent areas and the thalamus^16^. Further, we investigated MD correlates of mood states using by the Profile of Mood states^15^. However, we found robust significant negative correlation between motivational state (state vigor) and MD in the thalamus, putamen, pallidum, and contingent areas^15^. Also, there was no significant correlation between other mood states and MD. Therefore, although there are a variety of cognitive components in these traits of temperament and character inventory and states of Profile of Mood states, it seems that only MD of the limited areas show correlation with limited cognitive components of these traits and states and perhaps systemizing does not include these cognitive components. Future studies are needed to elucidate whether these (lack of correlation between MD and cognitive differences that do not include certain limited cognitive components) are applied to other states and traits and the mechanism behind this phenomenon.

Empathizing was positively correlated with the MD of the left postcentral gyrus and the Rolandic operculum areas. These regions overlap with areas of the key nodes of the mirror neuron system^51^. The involvement of these regions in empathizing may be consistent with the view that the mirror neuron system facilitates the understanding of the intentions of others and plays an important role in empathy^52,53^, as well as with the finding that reduced rGMV in these areas, which may be caused by advanced synaptic pruning, is associated with greater empathizing^8^. Depending on the situational context and the information available in the environment (such as the perceived fairness of another person or group membership and the similarities between oneself and others), empathic responses have been suggested to involve a co-recruitment of mirror neuron networks and regions involved in the theory of mind or mentalizing^54–56^.

Finally, cooperativeness showed significant positive correlation with MD in anatomical clusters that spread in and adjacent to the left lateral and medial parietal lobes, as well as in the anatomical cluster that spreads in and adjacent to the right lateral and medial parietal lobes, which were not observed in areas showing a significant positive correlation between empathizing and MD. Functional implications of these correlates are unclear because of lack of previous studies showing specific MD correlates in these areas, which are widespread and the contingent areas are associated with multiple functions^57^. However, one possibility is that posterior MD correlates around the posterior cingulate cortex (PCC) may relate to disrupted functions that inhibit prosociality, similar to the anterior MD correlates of cooperativeness, namely contentious interpersonal orientation, aggression, and anger. This is because the anterior part of PCC is functionally associated with negative emotions, such as anger, fear, and pain^58^. We have previously suggested an association between contentious interpersonal orientation and this area’s structural properties, given the correlation of PCC’s regional gray matter density with traits such as the hostile behaviors displayed by Type A personalities and competitive achievement motivation (i.e., the desire to manage and succeed in difficult tasks, directed at the pursuit of social prestige by defeating and outperforming others)^59,60^. Further, while a lack of serotonin plays a key role in aggression^61^, reduced serotonin in the PCC is associated with unfriendliness and greater social aggression in primates^62^. However, this is speculative and future studies are needed to better understand the impact of a greater MD in PCC among young normal adults.

Previous studies have investigated regional gray matter, white matter volume, and fractional anisotropy and resting state functional connectivity that is associated with empathizing, systemizing, and D score^7,8,23,63^. Each of these imaging measures can provide unique information about the brain, but MD measures reveal unique information that other techniques cannot provide. For example, the state and traits that are associated with cognitive components of motivation showed robust association with MD in the putamen and pallidum which play a key role in motivation^15,16^ and we previously showed that fatigue was positively correlated with MD in the basal ganglia; however, the amount of regional gray matter in these areas failed to show such associations^64^. As described above, cooperativeness showed a robust association with MD in and near areas that play key roles in emotional salience and anxiety^16^. But, to our knowledge, it is unknown whether different imaging techniques show association with cooperativeness in the same areas. Therefore, by using this unique measurement, we have elucidated the neural bases of empathizing.

In the present study, relatively small correlation coefficients were found between the mean MD values in the significance cluster and empathizing or systemizing (r < 0.11). In studies with large samples of young, normal individuals, relatively weak correlations (r < 0.2) between individual cognitive differences and neuroimaging measures are a universal phenomenon (i.e., N > several hundreds), regardless of the type of imaging measures^21,65–68^. This also holds true for associations between representative imaging measures and cognitive abilities, such as associations between gray matter volume or cortical thickness and general intelligence measures or working memory performance, and associations between white matter volume and processing speed. Therefore, the low correlation coefficients obtained in this study do not indicate a low relevance of the observed associations. It is noteworthy that in whole-brain imaging analyses with small samples, overfitting usually causes an extreme effect size overestimation^69^.

In conclusion, while increased MD is generally associated with decreased neural tissues and possibly function of an area, higher empathizing and cooperativeness was reflected by greater MD measurements of the areas in and adjacent to the left anterior and middle cingulate cortex, left lateral prefrontal cortex, and left insula. These areas mainly overlapped areas that play a key role in empathy and emotional salience. In addition, higher empathizing was reflected in greater MD of the left postcentral gyrus and left Rolandic operculum areas, which are overlapped with the areas of the mirror neuron system.

## Supplementary information

Supplementary online material
