# Supplementary material for: Empathizing associates with mean diffusivity
Source: Sci Rep. 2019 Jun 20;9:8856. doi: 10.1038/s41598-019-45106-1 (PMC6586867; doi:10.1038/s41598-019-45106-1)
Supplement: Supplementary file 1 — Supplementary online material [file 41598_2019_45106_MOESM1_ESM.pdf]

Supplemental online material

Empathizing associates with mean diffusivity

Hikaru Takeuchi[a], Yasuyuki Taki[a,b,c], Rui Nouchi[d,e,f], Ryoichi Yokoyama[g], Yuka Kotozaki[h], Seishu Nakagawa[i,j], Atsushi Sekiguchi[b], Kunio Iizuka[k], Yuki Yamamoto[i], Sugiko Hanawa[i], Tsuyoshi Araki[f], Carlos Makoto Miyauchi[l], Kohei Sakaki[i], Yuko Sassa[a], Takayuki Nozawa[m], Shigeyuki Ikeda[n], Susumu Yokota[a], Magistro Daniele[o,p], Ryuta Kawashima[a,f,n]

[a]*Division of Developmental Cognitive Neuroscience, Institute of Development, Aging and Cancer, Tohoku University, Sendai, Japan*

[b]*Division of Medical Neuroimaging Analysis, Department of Community Medical Supports, Tohoku Medical Megabank Organization, Tohoku University, Sendai, Japan*

[c]*Department of Radiology and Nuclear Medicine, Institute of Development, Aging and Cancer, Tohoku University, Sendai, Japan*

[d]*Creative Interdisciplinary Research Division, Frontier Research Institute for Interdisciplinary Science, Tohoku University, Sendai, Japan*

[e]*Human and Social Response Research Division, International Research Institute of Disaster Science, Tohoku University, Sendai, Japan*

[f] *Advanced Brain Science, Institute of Development, Aging and Cancer, Tohoku University, Sendai, Japan*

[g] *School of Medicine, Kobe University, Kobe, Japan*

[h]*Division of Clinical research, Medical-Industry Translational Research Center, Fukushima Medical University School of Medicine, Fukushima, Japan*

[i]*Department of Functional Brain Imaging, Institute of Development, Aging and Cancer, Tohoku University, Sendai, Japan*

[j]*Department of Psychiatry, Tohoku Pharmaceutical University, Sendai, Japan*

[k]*Department of Psychiatry, Tohoku University Graduate School of Medicine, Sendai, Japan*

[l] *Department of Language Sciences, Graduate School of Humanities, Tokyo Metropolitan University, 192-0397, Japan.*

[m]*Happiness Co-creation Society through "Ishin-Denshin" Intelligent Communications,Tokyo Institute of Technology,Tokyo, Japan*

[n]*Department of Ubiquitous Sensing, Institute of Development, Aging and Cancer, Tohoku University, Sendai, Japan*

[o]*School of Sport, Exercise, and Health Sciences, Loughborough University, Loughborough, England*

[p]*National Centre for Sport and Exercise Medicine (NCSEM), Loughborough University, Loughborough, England*

Corresponding author:

Hikaru Takeuchi

Division of Developmental Cognitive Neuroscience, IDAC, Tohoku University

4-1 Seiryo-cho, Aoba-ku, Sendai 980-8575, Japan

Tel/Fax: +81-22-717-7988

E-mail: takehi@idac.tohoku.ac.jp

Key words:

Empathizing; Systemizing; D score; mean diffusivity; diffusion tensor imaging

Supplemental Methods

Additional details of diffusion image acquisition. There are acquisitions for phase correction and for signal stabilization and these are not used as reconstructed images. MD and FA maps were calculated from the collected images using a commercially available diffusion tensor analysis package on the MR consol. This practice has been used in many of our previous studies [1-5]. Furthermore, the results of analyses using these images were congruent with those of previous studies in which other methods were used [6, 7], suggesting the validity of this method. These procedures involved correction for motion and distortion caused by eddy currents. Calculations were performed according to a previously proposed method [8]. These descriptions were mostly reproduced from our previous study using the same method [9].

Preprocessing of imaging data

Preprocessing and analysis of functional activation data were performed using SPM8 implemented in Matlab. Most of the following descriptions were reproduced from our previous study using the similar methods [9]. First, the skull in the mean b = 0 image of each participant was stripped as described previously [4]; using the resulting image, diffusion images were linearly aligned to the skull-stripped b = 0 image template

created previously [4] to assist with the following procedures.

Subsequently, using a previously validated two-step new segmentation algorithm of diffusion images and the previously validated diffeomorphic anatomical registration through exponentiated lie algebra (DARTEL)-based registration process that utilized the information of the FA signal distribution within the white matter tissue (for details, see ref [2]), all images, including gray matter segments [regional gray matter density (rGMD) map], white matter segments [regional white matter density (rWMD) map], and cerebrospinal fluid (CSF) segments [regional CSF density (rCSFD) map] of diffusion images, were normalized. The voxel size of these normalized images was 1.5 × 1.5 × 1.5 mm^3. In these processes, we used the template for the DARTEL process that we created in our previous study from subjects that participated in the same project (for details, see ref [2]).

Next, we created average images of normalized rGMD and rWMD images of all subjects whose diffusion imaging data were obtained in the pre-experiment. Subsequently, for the analyses of MD images from the normalized images of the (a) MD, (b) rGMD, and (c) rWMD maps, we created images where areas that were not strongly likely to be gray or white matter in our averaged normalized rGMD and rWMD images (defined by "gray matter tissue probability + white matter tissue probability <

0.99”) were removed (to exclude the strong effects of CSF on MD throughout analyses). These images were then smoothed (8 mm full-width half-maximum) and carried through to the second-level analyses of MD.

We did not use T1 weighted structural images for normalization and calculation of GMC and WMC maps for correction. This is because T1 weighted structural images and EPI images have apparent differences due to the distortion caused by 3T MRI and simply it is apparently not suited for the accurate and precise segmentation and normalization images of MD maps.

Supplemental Discussion

Limitations of this study

There was at least one limitation to this study, and this was identical to that of our previous studies as well as of other studies that use college cohorts [4, 10-13], is that we used young healthy subjects with a high educational background. Limited sampling of the full range of intellectual abilities and age is a common hazard when sampling from college cohorts [10]. Whether the findings from this cohort would also hold across the full range of population samples and a normal distribution must be determined with larger and more representative samples.

References

1. Takeuchi, H. et al. White matter structures associated with emotional intelligence: Evidence from diffusion tensor imaging. *Hum. Brain Mapp.* **34**, 1025-1034 (2013).
2. Takeuchi, H. et al. White matter structures associated with empathizing and systemizing in young adults. *Neuroimage* **77**, 222-236 (2013).
3. Takeuchi, H. et al. Training of Working Memory Impacts Structural Connectivity. *J. Neurosci.* **30**, 3297-3303 (2010).
4. Takeuchi, H. et al. White matter structures associated with creativity: Evidence from diffusion tensor imaging. *Neuroimage* **51**, 11-18 (2010).
5. Takeuchi, H. et al. Verbal working memory performance correlates with regional white matter structures in the fronto-parietal regions. *Neuropsychologia* **49**, 3466-3473 (2011).
6. Taki, Y. et al. Linear and curvilinear correlations of brain white matter volume, fractional anisotropy, and mean diffusivity with age using voxel-based and region of interest analyses in 246 healthy children. *Hum. Brain Mapp.* **34**, 1842-1856 (2013).
7. Barnea-Goraly, N. et al. White matter development during childhood and adolescence: a cross-sectional diffusion tensor imaging study. *Cereb. Cortex* **15**, 1848-1854 (2005).
8. Le Bihan, D. et al. Diffusion tensor imaging: concepts and applications. *Journal of Magnetic Resonance Imaging* **13**, 534-546 (2001).
9. Takeuchi, H. et al. Impact of videogame play on the brain's microstructural properties: Cross-sectional and longitudinal analyses. *Mol. Psychiatry* **21**, 1781-1789 (2016).

10. Jung, R.E. et al. Neuroanatomy of creativity. *Human brain mapping* **31**, 398-409 (2010).
11. Takeuchi, H. et al. Failing to deactivate: the association between brain activity during a working memory task and creativity. *Neuroimage* **55**, 681-687 (2011).
12. Takeuchi, H. et al. Regional gray matter volume of dopaminergic system associate with creativity: Evidence from voxel-based morphometry *Neuroimage* **51**, 578-585 (2010).
13. Song, M. et al. Brain spontaneous functional connectivity and intelligence. *Neuroimage* **41**, 1168-1176 (2008).
